# Asymptomatic SARS-CoV-2 Carriers: A Systematic Review and Meta-Analysis

**DOI:** 10.3389/fpubh.2020.587374

**Published:** 2021-01-20

**Authors:** Gopiram Syangtan, Shrijana Bista, Prabin Dawadi, Binod Rayamajhee, Lok Bahadur Shrestha, Reshma Tuladhar, Dev Raj Joshi

**Affiliations:** ^1^Shi-Gan International College of Science and Technology (SICOST), Tribhuvan University, Kathmandu, Nepal; ^2^Central Department of Microbiology, Tribhuvan University, Kathmandu, Nepal; ^3^Faculty of Science, School of Optometry and Vision Science (SOVS), University of New South Wales (UNSW), Sydney, NSW, Australia; ^4^Department of Infection and Immunology, Kathmandu Research Institute for Biological Sciences (KRIBS), Lalitpur, Nepal; ^5^Department of Microbiology and Infectious Diseases, B. P. Koirala Institute of Health Sciences, Dharan, Nepal

**Keywords:** asymptomatic, COVID-19, 2019-nCoV, pandemic, SARS-CoV-2, transmission

## Abstract

Asymptomatic cases of SARS-CoV-2 can be unknown carriers magnifying the transmission of COVID-19. This study appraised the frequency of asymptomatic individuals and estimated occurrence by age group and gender by reviewing the existing published data on asymptomatic people with COVID-19. Three electronic databases, PubMed, Embase, and Web of Science (WoS), were used to search the literature following the guidelines of Preferred Reporting Items for Systematic Reviews and Meta-Analysis (PRISMA). The study population for this review included asymptomatic individuals infected with SARS-CoV-2 reported in original articles published up to 30 April 2020. A random effects model was applied to analyze pooled data on the prevalence of asymptomatic cases among all COVID-19 patients and also by age and gender. From the meta-analysis of 16 studies, comprising 2,788 SARS-CoV-2 infected patients, the pooled prevalence according to the random effect size of asymptomatic cases was 48.2% (95% CI, 30–67%). Of the asymptomatic cases, 55.5% (95% CI, 43.6–66.8%) were female and 49.6% (95% CI, 20.5–79.1%) were children. Children and females were more likely to present as asymptomatic COVID-19 cases and could act as unknown carriers of SARS-CoV-2. Symptom-based screening might fail to identify all SARS-CoV-2 infections escalating the threat of global spread and impeding containment. Therefore, a mass surveillance system to track asymptomatic cases is critical, with special attention to females and children.

## Introduction

Coronaviruses are common respiratory pathogens causing illness in humans around the world (1). By the end of 2019, six different coronaviruses contagious to humans had been reported (2, 3). SARS-CoV-2 was identified as the seventh virus of the coronaviridae family causing infection to humans and the World Health Organization (WHO) announced “COVID-19” as a new disease on 11 February 2020 (4). In response to the rapid global spread of SARS-CoV-2, WHO declared COVID-19 as a Public Health Emergency of International Concern (PHEIC) on 30 January 2020 and a pandemic on 11 March 2020 (5). As of 26 May 2020, there were 5,508,904 confirmed cases of COVID-19 with 346,612 deaths globally (6).

The average incubation period of SARS-CoV-2 is 5–6 days but it can be up to 14 days (7). The clinical outcomes of COVID-19 can vary from asymptomatic to a mild to severe state. Common symptoms of COVID-19 include headache, fever, cough, fatigue, dyspnea, diarrhea, and even conjunctivitis, occasionally leading to severe SARS-like viral pneumonia, acute respiratory distress syndrome (ARDS), multi-organ dysfunction, and even death (8). In asymptomatic COVID-19 cases, people who test positive for SARS-CoV-2 nucleic acid by Real-time reverse transcriptase polymerase chain reaction (RT-PCR) do not develop symptoms (7). This population has two subpopulations: the pre-symptomatic and the true asymptomatic. Pre-symptomatic people are those with no symptoms who test positive for SARS-CoV-2 and later develop symptoms whereas true asymptomatic cases are people who test positive but never show any signs and symptoms (7, 9, 10). Adequate observations repeatedly taken for an extended period can help differentiate between asymptomatic and pre-symptomatic cases (11).

As asymptomatic COVID-19 cases do not present noticeable clinical symptoms, they frequently escape detection from public health surveillance systems and are challenging for possible preventive measures of infection control such as self-quarantine. Moreover, the cardinal route of SARS-CoV-2 transmission via aerosols exhaled by asymptomatic COVID-19 carriers during the act of breathing and speaking is well-documented (12–15) and cases of familial transmission through asymptomatic cases have been reported in different countries (12, 16–22). Undiagnosed asymptomatic individuals with COVID-19 were estimated to be up to 79% of SARS-CoV-2 infections in Wuhan, China (15). This highlights the critical role of asymptomatic cases in the progression of the ongoing pandemic. However, the demographic characteristics, clinical features and the actual prevalence of asymptomatic cases still remain elusive (23), which creates challenges for the prevention and containment of the COVID-19 pandemic. Therefore, this study was designed to evaluate the relative frequency of COVID-19 asymptomatic cases and estimate their occurrence by age group and gender by summarizing the existing published data and evidence on asymptomatic people with COVID-19.

## Methods

### Search Strategy and Data Sources

Three electronic databases, PubMed, Embase, and Web of Science (WoS), were used to search for articles following the PRISMA guidelines (24). The PRISMA checklist (Supplementary Table 1) was followed in conducting this study. Medical Subject Headings (MeSH) and key words, using “OR” and “AND,” were used to search published articles in the electronic databases. The following search terms were used: “Novel coronavirus 2019,” or “2019 nCoV,” or “COVID-19,” or “Asymptomatic cases/carriers of COVID-19,” or “Asymptomatic infections with COVID-19,” and “SARS-CoV-2.” Articles published in English between 1 January 2020 and 30 April 2020 were included.

### Selection Criteria

Authors GS, PD, SB and DRJ evaluated the search results and independently determined the eligibility of studies. Any dissonance regarding inclusion and exclusion of the studies was resolved by discussion between the authors. Any discrepancy during the review of full articles was resolved with a majority vote.

### Eligibility Criteria

The inclusion criteria set for the selection of study articles were:

- study population: patients with SARS-CoV-2 infections confirmed by RT-PCR but without symptoms at time of screening
- study design: prospective/retrospective cohort study, case-control studies, and case reports published in peer-reviewed journals.

The exclusion criteria were:

- studies without a proportion of asymptomatic SARS-CoV-2 infected patients
- review and opinion articles, published protocols, meta-analyses of primary data, editorials, and cases published in languages other than English.

### Study Selection

The title and abstract of the articles selected from the initial search strategy were scrutinized to screen for relevant articles. Then, the full text of relevant articles was inspected using the inclusion and exclusion criteria. Studies that reported the proportion of asymptomatic SARS-CoV-2 infected patients were included for quantitative analysis.

### Data Extraction

Elements recorded from the screened articles included first author, type of study, publishing institution, date of publication, site of study, sample size (Table 1), age and gender of patients, and ratio of asymptomatic and symptomatic cases (Supplementary Table 3).

**Table 1:** Characteristics of the studies included for meta-analysis.

**S.N**.	**Study ID**	**Study period**	**Country of study**	**Total number of confirmed cases of COVID-19**	**Asymptomatic cases among confirmed cases [*N* (%)]**
1	(25)	April 2020	Homeless Shelter in Boston, USA	147	129 (87.8%)
2	(26)	January 16 to February 8, 2020	China	728	94 (12.92%)
3	(27)	January 28 to February 9, 2020	The Second Hospital of Nanjing, China	24	24 (100%)
4	(28)	March 2020	Nursing Facility-King County, USA	23	13 (56.52%)
5	(29)	January 28 to February 26,2020	The Wuhan Children's Hospital, China	171	39 (22.81%)
6	(30)	January 1 to February 23, 2020	Renmin Hospital of Wuhan University, China	58	58 (100%)
7	(31)	February,2020	Diamond Princess cruise ship, Yokohama, Japan	634	328 (51.74%)
8	(23)	February 6, 2020	Japan	13	4 (30.8%)
9	(32)	January 10 to March 10, 2020	China	26	26 (100%)
10	(33)	January 17 to March 1, 2020	Three hospitals in Zhejiang province, China	36	10 (28%)
11	(34)	January 26 to March 6, 2020	People's hospital of Dafou county, China	83	18 (21.7%)
12	(35)	March 22 to April 4, 2020	Two hospitals in New York, USA	33	29 (87.9%)
13	(36)	January to March 2020	Chongqing Public Health Medical Center, Chongqing, China	167	20 (11.98%)
14	(7)	January 20 to February 10, 2020	China	262	13 (4.9%)
15	(37)	February 23, 2020	Third People's Hospital of Shenzhen, China	55	55 (100%)
16	(38)	March 4, 2020	Shanghai Public Health Clinic Center, China	328	13 (3.96%)
Total				2,788	873

### Outcome Measurements

The primary finding of this study was the prevalence of asymptomatic cases among total SARS-CoV-2 infected individuals. The secondary outcome measures included prevalence of asymptomatic cases by gender and age group and prevalence of true asymptomatic cases within total asymptomatic cases.

### Risk of Bias (Quality) Assessment in Individual Studies

The potential risk of bias in each included study was assessed using the Newcastle–Ottawa Scale (NOS) for observational studies (case-control, cohort, and cross-sectional studies) (39). Studies were graded out of 10 points (stars) as shown in Supplementary Table 2. The mean score of two reviewers (GS, SB) was considered to make the decision. Any variation in individual scores was checked and resolved by a third author (DRJ). As there is no standard cut-off score, we included all the studies with an arbitrary score value ≥ 6. This quality assessment was performed to assess the systematic error and external validity of studies and ultimately reduce the risk of biases.

### Publication Bias Assessment

The potential publication bias was assessed by plotting a standard error and precision with Logit event rate as funnel plots. The absence of publication bias among the included studies was also confirmed by Begg's (Begg and Mazumdar Rank Correlation) and Egger's test (regression intercept) with *p* > 0.05 indicating no publication bias (40, 41).

### Data Analysis

Comprehensive Meta-Analysis version 3 statistical software (https://www.meta-analysis.com/) was used for the statistical analysis. Percentages were calculated to describe the distributions of categorical variables. The prevalence of asymptomatic cases of SARS-CoV-2 infection was expressed as a proportion with a 95% confidence interval using a random effects model and was presented as a Forest plot. The Cochran *Q* test and inconsistency index (*I*^2^) were used to detect heterogeneity among studies, with a *p* < 0.05 indicating significant heterogeneity. *I*^2^ values of < 25, 25–75, and >75% indicate low, moderate, and high heterogeneity respectively (42).

### Subgroup Analysis

Subgroup analysis was performed in targeted patient age groups (children, adults, and elderly), gender groups (male and female) and clinical outcome of asymptomatic cases (pre-symptomatic and true asymptomatic). Individuals 18 years and younger were considered children as recognized by the United Nations Convention on the Rights of the Child (43). Asymptomatic cases without typical symptoms associated with COVID-19 during the time of hospitalization were categorized as “true asymptomatic cases” and cases not showing symptoms during a 14-day incubation period but later exhibiting symptom were categorized as “pre-symptomatic cases” (44–46). Only studies that reported age, gender and true cases of asymptomatic COVID-19 patients were included in the subgroup analysis of age, gender, and clinical outcome of asymptomatic cases.

## Results

### Study Selection

**Figure 1:**
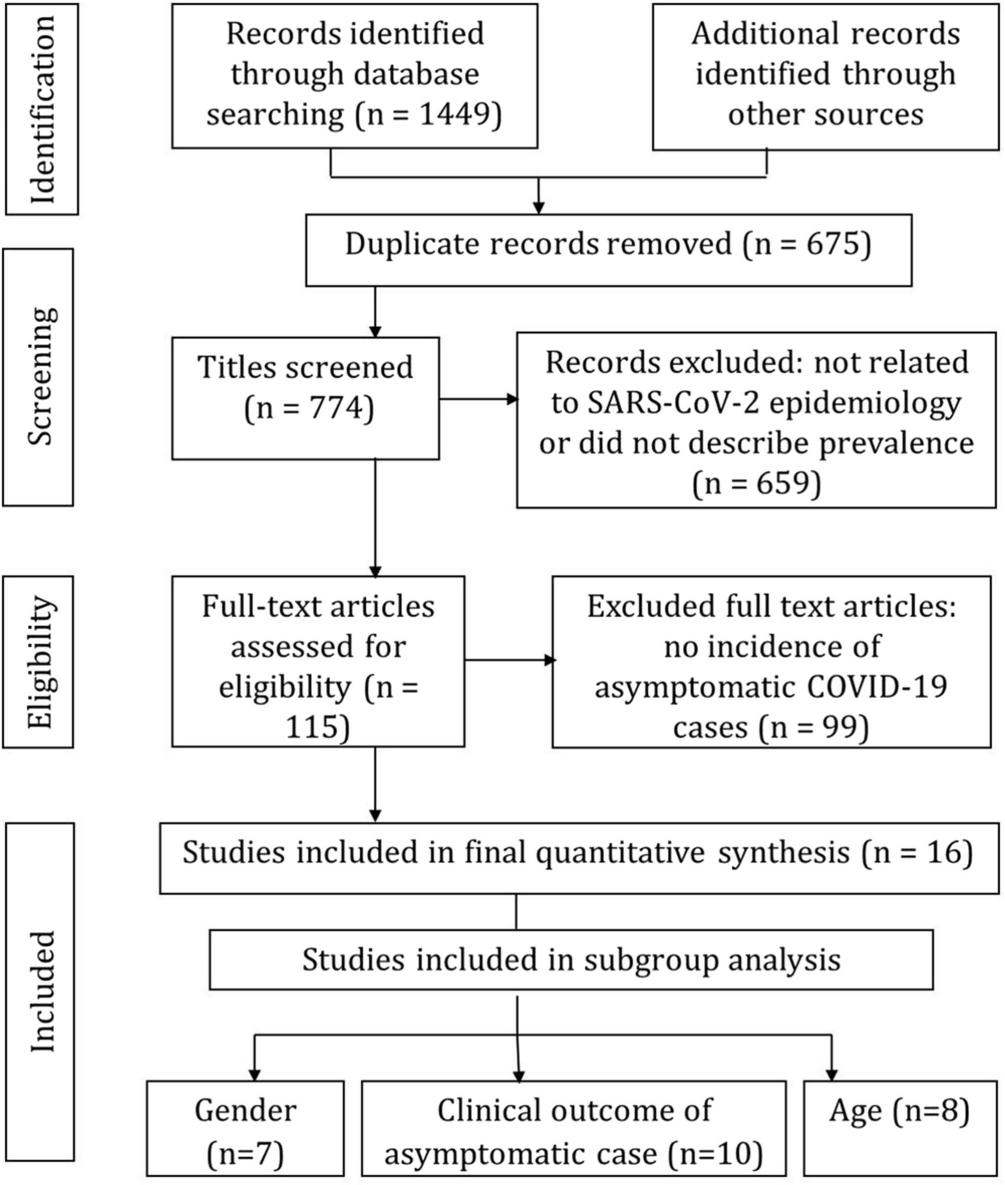
PRISMA flow diagram of screening process for the selection of studies for meta-analysis.

A total of 1,449 potentially relevant articles were retrieved through the PRISMA search strategy. After removing 675 duplicate articles, the remaining 774 articles were further investigated by title and abstract and 115 articles were included for full text assessment. As shown in the PRISMA flow diagram (Figure 1), of the 115 full text assessed articles, 16 (7, 23, 25–38) were included for the quantitative meta-analysis and 99 were excluded due to the absence of information about the incidence of asymptomatic COVID-19 cases.

### Study Characteristics

This systematic review included 16 studies published between 1 January and 30 April 2020. Most studies were from China (*n* = 11), two were from Japan and three were from the USA, with a total of 2,788 SARS-CoV-2 infected patients (Table 1). This review integrated results from both cross-sectional and observational cohort studies.

### Demographic Characteristics and Clinical Manifestation of COVID-19 Patients

Of the SARS-CoV-2 infected patients, 54.4% were male and 45.6% were female. Of the SARS-CoV-2 infected patients, 42.5% were children ( ≤ 18 years), 22.8% were adults (19–50 years), and 34.7% were elderly (≥51 years). Regarding clinical characteristics, 873 were asymptomatic cases and 1,915 were symptomatic cases with varying clinical symptoms of fever, cough, sore throat, myalgia, fatigue, headache, and dyspnea. Demographic and clinical features of COVID-19 patients are summarized in Supplementary Table 3.

### Meta-Analysis of Asymptomatic Cases of COVID-19

**Figure 2:**
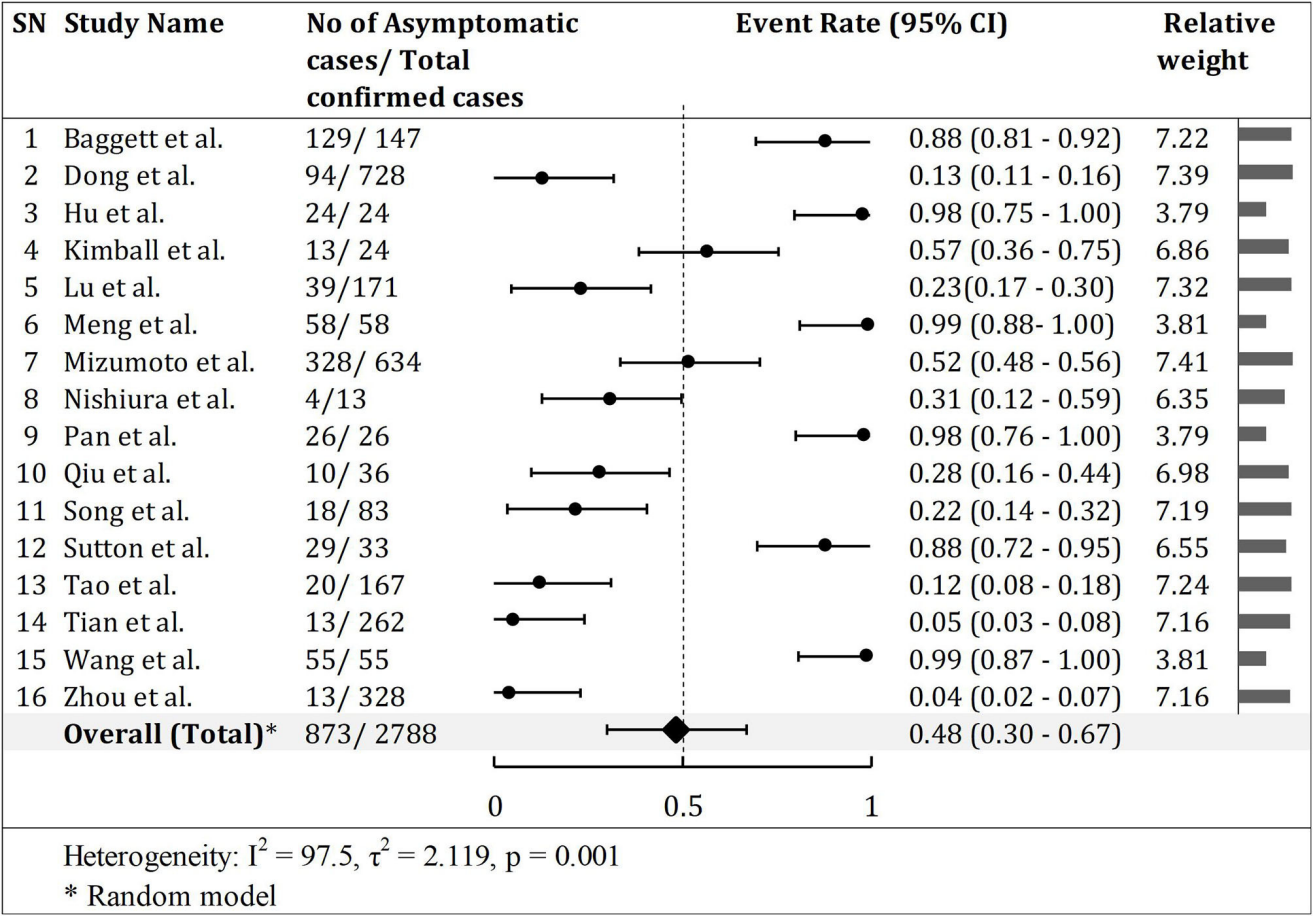
Asymptomatic infection rate in COVID-19 patients. Half of the studies (8) had event rate (ratio) of asymptomatic cases over 0.5. However, the forest shows overall asymptomatic event ratio 0.48 (48.2%).

The pooled prevalence of asymptomatic cases of COVID-19 from a total of 2,788 confirmed SARS-CoV-2 infected patients in 16 studies was 48.2% (95% CI, 30–67%), with significant heterogeneity noted among studies (*p* < 0.001; *I*^2^ = 97.5; Figure 2). However, the actual proportion of asymptomatic cases was 31.3% (873/2,877) as shown in Table 1.

### Subgroup Analysis

#### Gender

**Figure 3:**
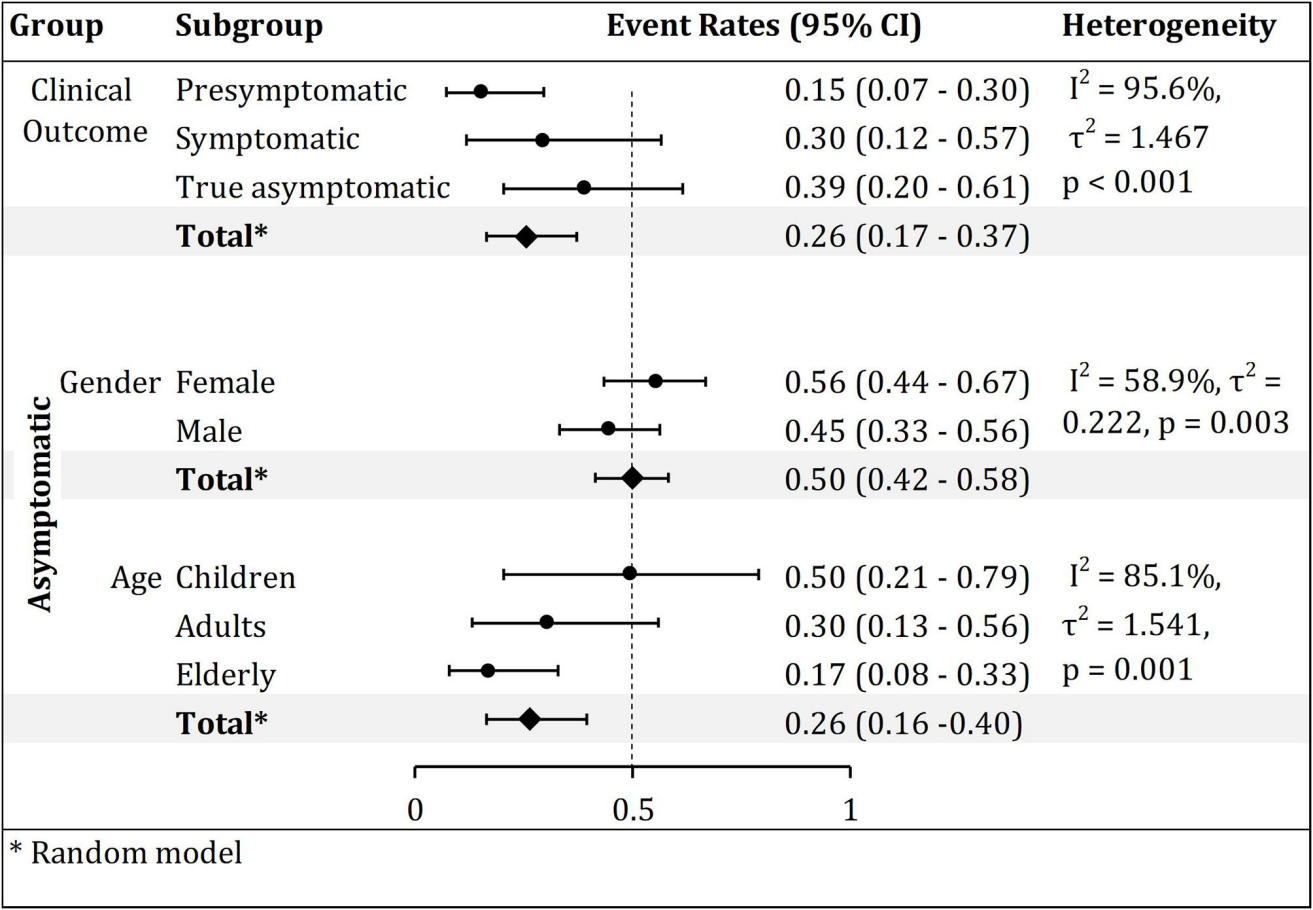
Subgroup analysis of pooled clinical outcome, and gender and age-based prevalence of asymptomatic COVID-19 cases. The forest plot of event rates (ratios) showed higher prevalence of asymptomatic (0.39), female (0.56), and children (0.50) COVID 19 patients.

Of the 223 asymptomatic COVID-19 cases in seven studies, 55.5% (95% CI, 43.6–66.8%) were female and 44.5% (95% CI, 33.2–56.4%) were male (Supplementary Figure 1). Although there was no significant subgroup difference between males and females (*p* = 0.199) (data not shown), moderate heterogeneity was noted among the studies (*p* < 0.001; *I*^2^ = 58.9%; Figure 3).

#### Age

In a subgroup analysis of eight studies, among 318 asymptomatic cases of COVID-19, 49.6% (95% CI, 20.5–79.1%) were children ( ≤ 18 years), 30.3% (95% CI, 13–56%) were adults (19–50 years) and only 16.9% (95% CI, 7.8–32.9%) were elderly (≥51 years) (Supplementary Figure 2). Despite insignificant subgroup difference by age group (*p* = 0.142), there was significant high heterogeneity among studies (*p* < 0.001; *I*^2^ = 85.1%; Figure 3).

### Clinical Outcome of Asymptomatic Cases of COVID-19

The pooled prevalence of true asymptomatic cases of COVID-19 from a total of 1,277 confirmed SARS-CoV-2 infected patients in 10 studies was 39% (95% CI, 20.4–61.4%; Supplementary Figure 3). The pooled prevalence of pre-symptomatic cases was 15.3% (95% CI, 7.2–29.6%). There was significant high heterogeneity among the studies (*p* < 0.001; *I*^2^ = 95.6%; Figure 3).

### Publication Bias

The funnel plot (Supplementary Figure 4) showed symmetry, demonstrating the absence of publication bias among the included studies. The Begg's (Begg and Mazumdar rank Correlation) and Egger's (regression intercept**)** tests also confirmed there was no visible evidence of publication bias among the included studies for prevalence of asymptomatic COVID-19 cases. Kendall's tau with continuity correction in the Begg and Mazumdar rank Correlation [*p* (2-tailed) = 0.0884] and Egger's regression intercept [*p* (2-tailed) = 0.7472] were insignificant. (Supplementary Table 4).

## Discussion

The pooled prevalence of asymptomatic cases of COVID-19 from this meta-analysis of 2,788 infected patients was 48.2% as estimated by random effect size. In a case series of 78 patients from 26 transmission clusters in Wuhan, China, a similar estimate (42.3%) of asymptomatic carriers in SARS-CoV-2 infected individuals was reported (22). However, considering the basic reproduction number (*R*_0_) of 2.5, a slightly lower asymptomatic SARS-CoV-2 infection rate of 35% was estimated by the US Centers for Disease Control and Prevention (CDC) (47). This indicates a large number of asymptomatic cases of COVID-19 are in the community seeding potential outbreaks which requires vigilant control strategies to prevent future outbreaks. Thus, mask wearing, hand washing, physical distancing, and extensive testing, followed by quarantine of infected asymptomatic individuals, are essential to contain the rapid spread of SARS-CoV-2 locally and globally (48).

Due to the high prevalence of asymptomatic cases, it is important to seek reasons for SARS-CoV-2 infection in people without visible symptoms (49). Some studies have highlighted that the cross-reactive T-cell response with exposure to other coronaviruses might have contributed to the asymptomatic phenotype in SARS-CoV-2 infected individuals (50–53). This is supported by the 67% homology in the sequences of epitope between common cold coronaviruses and SARS-CoV-2, and an increased number of CD4+ T cells in asymptomatic patients compared to symptomatic patients (54, 55).

SARS-CoV, SARS-CoV-2, and MERS, despite all belonging to the genus *Betacoronavirus*, differ in their disease transmission and clinical features (56–58). SARS and MERS were mainly associated with nosocomial spread whereas SARS-CoV-2 is being widely disseminated both in the community and hospital environments (59). The prevalence of asymptomatic infection was comparatively lower in MERS and SARS, at about 9.8% (60) and 13% (61), respectively. The higher asymptomatic infection of SARS-CoV-2 may be related to the high replication efficiency of this virus, which is three times greater than SARS-CoV (62, 63). Thus, SARS-CoV-2 rapidly disseminates to the pharynx and sheds prior to the activation of the innate immune response and presence of symptoms (64). Another contributing factor could be a less significant induction of host interferon and pro-inflammatory response, which also distinguishes SARS-CoV-2 from other SARS-CoV strains (64). The proportion of asymptomatic cases of COVID-19 has gradually increased since the first reported outbreak in Wuhan, China (65). This may be because of decreased pathogenicity of SARS-CoV-2 in the process of the long chain of transmission (36).

The communicable period of asymptomatic cases of COVID-19, defined as the interval between the 1st day of positive nucleic acid tests to the 1st day of continuous negative tests, can last for a month (27). Thus, asymptomatic cases of COVID-19 may carry the virus for a long period of time and spread it unknowingly. Potential SARS-CoV-2 transmission from asymptomatic individuals has been documented in many studies (19–21, 27). A high viral load was detected when SARS-CoV-2 was assessed in the upper respiratory specimens of asymptomatic, pre-symptomatic and symptomatic patients, suggesting potential for transmission regardless of symptoms (66). The biological evidence for this was supported by a study in a skilled nursing facility where infectious SARS-CoV-2 culture was grown from the upper respiratory tract specimens of pre-symptomatic and asymptomatic patients 6 days before the development of COVID-19 related symptoms (67).

Of the total SARS-CoV-2 infected patients included in this meta-analysis, 54.4% were male and 45.6% were female. This implies that men were more susceptible to SARS-CoV-2 infection than women. The COVID-19 pandemic has shown a striking gender bias with more cases and a higher mortality rate in men than in women (68). Data from the WHO and Chinese studies indicate that ~1.7% of females who contracted SARS-CoV-2 have died compared to 2.8% of males (69). Increased male susceptibility might be explained by biological and behavioral factors (70). Biological factors include men's high level of testosterone that inhibits antibody production, and the presence of angiotensin-converting enzyme 2 (ACE2) receptors (cell receptors which play an essential role in SARS-CoV-2 entry) that facilitate viral replication (71). Similarly, social, behavioral, and lifestyle factors include men's higher rates of smoking and alcohol consumption, and low level of hand-washing practices (72), although there is no clear evidence these behavioral factors have any impact on COVID-19 transmission. In general, women have a stronger immune response against viral infections than men (73), which possibly gives women some immunological protection (74). Gender-based hormones also regulate and influence aspects of viral entry including expression and activity of ACE2 and trans-membrane serine protease 2 (TMPRSS2) (69). The gene expression of ACE2, a, together with TMPRSS2 may also influence intensity of SARS-CoV-2 infection.

Subgroup analysis of asymptomatic COVID-19 patients revealed that children are the largest age group of asymptomatic carriers of SARS-CoV-2 (49.6%), followed by adults (30.3%), and the elderly (16.9%). A higher incidence of asymptomatic cases of COVID-19 in children can be related to both exposure and host factors. It is known that children's immune systems are not well-developed, and the maturity and binding ability of ACE2 in children may be lower than in young adults (75). However, children often experience numerous viral infections, and their repeated viral exposure is anticipated to aid their immune response to SARS-CoV-2 (76). Meanwhile, elderly people with a weakened immune system (77, 78) are less likely to be asymptomatic carriers. In contrast, adults who most likely have a stronger immune system can be infected and remain asymptomatic carriers (79). However, a detailed mechanism for the differences in asymptomatic manifestation of SARS-CoV-2 among these three age groups (children, adults and the elderly) is yet to be explored.

The pooled prevalence of asymptomatic SARS-CoV-2 infection was observed to be higher in females (55.5%) than in males (44.5%) from subgroup analysis. Aligning with our results, more women (66.7%) were observed to be asymptomatic among 78 close contacts of COVID-19 patients in Wuhan, China (22). Although there was no significant subgroup difference by gender, higher prevalence of asymptomatic cases in females could arise from host factors in women (80). Compared to men, women exhibit stronger innate, cellular, and humoral immune responses due to increased activation effects of female sex hormones and the presence of immune response genes on sex chromosomes (81). These enhanced innate and adaptive immune responses could have contributed to the increased asymptomatic SARS-CoV-2 infection in women.

Of the total asymptomatic SARS-CoV-2 cases included in the subgroup analysis, 39% cases did not have any symptoms while 15.3% were pre-symptomatic cases with mild COVID-19 related symptoms including fever, malaise and cough 3 days after a RT-PCR nucleic acid test positive (28, 30). This data inferred that a small fraction of asymptomatic cases may eventually become symptomatic. Thus, in order to evaluate the actual prevalence of true COVID-19 asymptomatic cases, longitudinal observations of cases for at least 14 days is recommended.

A recent review reported that as the surveillance and contact tracing of MERS progressed over time, the rate of asymptomatic MERS infected patients increased to 28.6% (60). The increase in asymptomatic infection of MERS was inversely proportional to the case fatality rate (82). This clearly demonstrates the importance of mass surveillance and contact tracing in the detection of asymptomatic SARS-CoV-2 infected individuals in the community and hospitals to reduce the disease fatality rate and dissemination of COVID-19. Transmission of COVID-19 by asymptomatic people is the weakness of COVID-19 control and prevention strategies (17). Hence, mass testing for SARS-CoV-2 in asymptomatic people should be increased especially in mass living conditions such as prisons, hospitals, camps, nursing, and elder care facilities. To contain the rapid spread of COVID-19, screening and testing of SARS-CoV-2 asymptomatic carriers along with other control strategies must be prioritized at the community level.

The strengths of this study include comprehensive analysis of 16 studies adding precision to the estimation of the prevalence of asymptomatic SARS-CoV-2 infection. Subgroup analysis revealed that children and females were the most likely asymptomatic carriers of SARS-CoV-2, highlighting the necessity of focusing on these target populations to control the pandemic. An additional strength of this study was the evaluation of asymptomatic and pre-symptomatic cases which helped understand the dynamics of this pandemic.

Despite strong findings, this study has some limitations. As asymptomatic patients are very likely to be unnoticed and not detected, the pooled prevalence rate in this study might be under-reported. Moreover, the majority of the studies in this meta-analysis were from China, with few ethnic groups other than Chinese, because the meta-analysis was conducted only 3 months after the pandemic started. More comprehensive studies including a wider range of ethnicities from global communities are needed to provide a more precise estimate of the prevalence of asymptomatic SARS-CoV-2 cases in a broader context.

## Conclusion

About half of the SARS-CoV-2 infected patients were asymptomatic at the time of screening. Despite the limitation, the findings of this study highlight that females and children were the predominant groups without symptoms of COVID-19. However, in general, asymptomatic infections can occur in any age range and either gender. As asymptomatic carriers play a critical role in the spread of the COVID-19 pandemic, understanding the actual prevalence of asymptomatic cases is important for setting control measures in both the community and health care centers. The high prevalence of asymptomatic COVID-19 cases suggests that screening based only on symptoms might fail to identify a large proportion of SARS-CoV-2 infections, escalating the threat of rapid spread. Thus, mask wearing, extensive testing for identification, and the quarantine of infected asymptomatic individuals are essential to curb this pandemic.

## Data Availability Statement

The original contributions presented in the study are included in the article/Supplementary Material, further inquiries can be directed to the corresponding author/s.

## Author Contributions

BR conceived the idea and BR, GS, and DJ designed the study. GS, PD, and SB reviewed the literature, extracted data, performed data analysis, and drafted the manuscript. DJ, BR, LS, and RT revised and reviewed the manuscript. BR and GS substantially revised the manuscript. DJ supervised the overall data analysis and writing. All the authors read and approved the final manuscript.

## Conflict of Interest

The authors declare that the research was conducted in the absence of any commercial or financial relationships that could be construed as a potential conflict of interest.

## Abbreviations

ACE2: Angiotensin-Converting Enzyme 2
ARDS: Acute Respiratory Distress Syndrome
CDC: Centers for Disease Control and Prevention (US)
COVID-19: Corona Virus Disease 2019
MERS-CoV: Middle East Respiratory Syndrome Coronavirus
MeSH: Medical Subject Headings
2019-nCoV: 2019 Novel Coronavirus
PHEIC: Public Health Emergency of International Concern
PRISMA: Preferred Reporting Items for Systematic Reviews and Meta-Analysis
RT-PCR: Reverse Transcriptase Polymerase Chain Reaction
SARS-CoV: Severe Acute Respiratory Syndrome Coronavirus
SARS-CoV-2: Severe Acute Respiratory Syndrome Coronavirus 2
USA: United States of America
WHO: World Health Organization.
