# Supplementary material for: Asymptomatic SARS-CoV-2 Carriers: A Systematic Review and Meta-Analysis
Source: Front Public Health. 2021 Jan 20;8:587374. doi: 10.3389/fpubh.2020.587374 (PMC7855302; doi:10.3389/fpubh.2020.587374)
Supplement: Supplementary file 1 [file Data_Sheet_1.PDF]

<u>Supplementary Material-1 (SM-1)</u>

PRISMA Check List

Undetected asymptomatic SARS-CoV-2 carriers: A systematic review and meta-analysis

Supplementary Material (SM) includes:
Number of Pages: 5
Number of Table: 1

Table S1: PRISMA Checklist for meta-analysis of prevalence of COVID-19 Asymptomatic patients

S.N.	Section/Topic	Checklist item	Reported on page
TITLE			
1	Title	Identify the report as a systematic review, meta-analysis, or both.	1
ABSTRACT			
2.	Structured summary	Provide a structured summary including, as applicable: background; objectives; data sources; study eligibility criteria, participants, and interventions; study appraisal and synthesis methods; results; limitations; conclusions and implications of key findings.	1-2 (Abstract)
INTRODUCTION			
3.	Rationale	Describe the rationale for the review in the context of what is already known.	3 (Introduction, paragraph3)
4.	Objectives	Provide an explicit statement of questions being addressed with reference to participants, interventions, comparisons, outcomes, and study design (PICOS).	3 (Introduction, paragraph3)
METHODS			
5.	Protocol and registration	Indicate if a review protocol exists, if and where it can be accessed (e.g., Web address), and, if available, provide registration information including registration number.	4 (Methods, paragraph 1) Registration N/A
6.	Eligibility criteria	Specify study characteristics (e.g., PICOS, length of follow-up) and report characteristics (e.g., years considered, language, publication status) used as criteria for eligibility, giving rationale.	4 (Methods, paragraph 3)
7.	Information sources	Describe all information sources (e.g., databases with dates of coverage, contact with study authors to identify additional studies) in the search and date last searched.	4 (Methods, paragraph 1)

8.	Search	Present full electronic search strategy for at least one database, including any limits used, such that it could be repeated.	4 (Methods, paragraph 2)
9.	Study selection	State the process for selecting studies (i.e., screening, eligibility, included in systematic review, and, if applicable, included in the meta-analysis).	4-5(Methods, paragraph 4)(Figure 1)
10.	Data collection process	Describe method of data extraction from reports (e.g., piloted forms, independently, in duplicate) and any processes for obtaining and confirming data from investigators.	4 (Methods, paragraph 2)
11.	Data items	List and define all variables for which data were sought (e.g., PICOS, funding sources) and any assumptions and simplifications made.	5 (Methods, paragraph 5)
12.	Risk of bias in individual studies	Describe methods used for assessing risk of bias of individual studies (including specification of whether this was done at the study or outcome level), and how this information is to be used in any data synthesis.	5 (Methods, paragraph 7) NOS Table S2 (SI file)
13.	Summary measures	State the principal summary measures (e.g., risk ratio, difference in means).	5 (Methods, paragraph 6)
14.	Synthesis of results	Describe the methods of handling data and combining results of studies, if done, including measures of consistency (e.g., I^2) for each meta-analysis.	6 (Methods, paragraph 9)
15.	Risk of bias across studies	Specify any assessment of risk of bias that may affect the cumulative evidence (e.g., publication bias, selective reporting within studies).	5 (Methods, paragraph 8)
16.	Additional analyses	Describe methods of additional analyses (e.g., sensitivity or subgroup analyses, meta-regression), if done, indicating which were pre-specified.	6 (Methods, paragraph 10)

RESULTS			
17.	Study selection	Give numbers of studies screened, assessed for eligibility, and included in the review, with reasons for exclusions at each stage, ideally with a flow diagram.	6 (Results, paragraph 1) (Fig. 1)
18.	Study characteristics	For each study, present characteristics for which data were extracted (e.g., study size, PICOS, follow-up period) and provide the citations.	7 (Results, paragraph, 2) (Table 1; SI, Table S3)
19.	Risk of bias within studies	Present data on risk of bias of each study and, if available, any outcome level assessment (see item 12).	(SI, Table S2)
20.	Results of individual studies	For all outcomes considered (benefits or harms), present, for each study: (a) simple summary data for each intervention group (b) effect estimates and confidence intervals, ideally with a forest plot.	7 (Results, paragraph, 3 and 4) (Figure 2, Figure 3)
21.	Synthesis of results	Present results of each meta-analysis done, including confidence intervals and measures of consistency.	7-8 (Results, paragraph, 4, 5, 6, 7) (Figure 3)
22.	Risk of bias across studies	Present results of any assessment of risk of bias across studies (see Item 15).	8 (Results, Paragraph 8) (SI, Figure S4, Table S4)
23.	Additional analysis	Give results of additional analyses, if done (e.g., sensitivity or subgroup analyses, meta-regression [see Item 16]).	7-8 (Results, paragraph, 5, 6 and 7) (Figure 3)
DISCUSSION			
24.	Summary of	Summarize the main findings including the strength of evidence for each main	8,10-11 (Discussion,

	evidence	outcome; consider their relevance to key groups (e.g., healthcare providers, users, and policy makers).	paragraph, 1, 5, 6 and 8)
25.	Limitations	Discuss limitations at study and outcome level (e.g., risk of bias), and at review-level (e.g., incomplete retrieval of identified research, reporting bias).	11 (Discussion, paragraph, 10)
26.	Conclusions	Provide a general interpretation of the results in the context of other evidence, and implications for future research.	11-12 (Conclusion, paragraph 1)
FUNDING			
27.	Funding	Describe sources of funding for the systematic review and other support (e.g., supply of data); role of funders for the systematic review.	N/A
