# Supplementary material for: Asymptomatic SARS-CoV-2 Carriers: A Systematic Review and Meta-Analysis
Source: Front Public Health. 2021 Jan 20;8:587374. doi: 10.3389/fpubh.2020.587374 (PMC7855302; doi:10.3389/fpubh.2020.587374)
Supplement: Supplementary file 2 [file Data_Sheet_2.PDF]

Supplementary Material – 2 (SM – 2)

Supplementary Material (SM) includes -

Number of Pages: 8

Number of Tables: 3

Number of Figures: 4

Table S2. Newcastle-Ottawa Scale for quality assessment of included observational studies

S.N.	Study Name	Methodological quality (max 5 stars)	Comparability (max 2 stars)	Outcome measures and analysis (max 3 stars)	Total score
1.	Baggett et al. 2020	****	**	**	8
2.	Dong et al. 2020	****	**	***	9
3.	Hu et al. 2020	***	**	***	8
4.	Kimball et al. 2020	****	**	***	9
5.	Lu et al. 2020	***	**	**	7
6.	Meng et al. 2020	***	**	***	8
7.	Mizumoto et al. 2020	****	**	***	9
8.	Nishiura et al. 2020	****	*	***	8
9.	Pan et al. 2020	***	**	***	8
10.	Qiu et al. 2020	*****	*	***	9
11.	Song et al. 2020	**	**	**	6
12.	Sutton et al. 2020	****	*	***	8
13.	Tao et al. 2020	****	**	***	9
14.	Tian et al. 2020	****	**	***	9
15.	Wang et al. 2020	***	**	***	8
16.	Zhou et al. 2020	***	**	***	8

*, One score

Table S3. Demographic and clinical characteristics of COVID-19 patients from the studies included in the meta-analysis

S.N.	Study Name*	Gender			Age			Clinical observation	
		Male	Female	Other	Children (≤18 years)	Adult (19-50 yrs)	Elderly (≥51 yrs)	Symptomatic	Asymptomatic
1.	Baggett et al. (2020)	124	22	1	0	51	96	18	129
2.	Dong et al. (2020)	418	310		728	0	0	634	94
3.	Hu et al. (2020)	8	16		5	9	10	0	24
4.	Kimball et al. (2020)	7	16		0	23	0	10	13
5.	Lu et al. (2020)	104	67		171	0	0	132	39
6.	Meng et al. (2020)	26	32		0	48	10	0	58
7.	Mizumoto et al. (2020)	321	313		6	77	551	306	328
8.	Nishiura et al. (2020)	-	-		-	-	-	9	4
9.	Pan et al. (2020)	16	10		-	-	-	0	26
10.	Qiu et al. (2020)	23	13		36	0	0	26	10
11.	Song et al. (2020)	-	-		-	-	-	65	18
12.	Sutton et al. (2020)	0	33		-	-	-	4	29
13.	Tao et al. (2020)	-	-		7	97	63	147	20
14.	Tian et al. (2020)	127	135		11	203	48	249	13
15.	Wang et al. (2020)	22	33		15	18	22	0	55
16.	Zhou et al. (2020)	-	-		-	-	-	315	13
	**Total**							**1915**	**873**

*References for all included studies are listed in the 'References' of the manuscript.

Table S4. Begg's and Egger's test for assessment of public bias in the meta-analysis

Begg and Mazumdar rank Correlation	
Kendall's S statistic (P-Q)	30
Kendall's tau without continuity correction	
Tau	
Z-value for tau	0.25
P-value (1-tailed)	1.3507
P - value (2- tailed)	0.0884
Kendall's tau with continuity correction	
Tau	0.2417
Z-value for tau	1.3057
P-value (1-tailed)	0.0958
P - value (2- tailed)	0.1917

Egger's regression intercept	
Intercept	0.8079
Standard Error	2.4575
95% lower limit (2- tailed)	-4.4529
95% upper limit (2- tailed)	6.0787
t-value	0.3736
Df	14
P - value (1-tailed)	0.3736
P - value (2-tailed)	0.7472

Figures

SN	Study Name	Sub-group	Event Rate (95% CI)	Relative weight
3	Hu et al.	Female	0.67 (0.46 - 0.82)	14.90
6	Meng et al.		0.55 (0.42 - 0.67)	21.12
9	Pan et al.		0.39 (0.22 - 0.58)	15.89
11	Song et al.		0.39 (0.20 - 0.62)	13.35
12	Sutton et al.		0.98 (0.78 - 1.00)	2.65
15	Wang et al.		0.60 (0.47 - 0.72)	20.68
16	Zhou et al.		0.54 (0.28 - 0.78)	11.41
	Overall (Female)*		0.56 (0.44 - 0.67)	
3	Hu et al.	Male	0.33 (0.18 - 0.54)	14.90
6	Meng et al.		0.45 (0.33 - 0.58)	21.12
9	Pan et al.		0.62 (0.42 - 0.78)	15.89
11	Song et al.		0.61 (0.38 - 0.80)	13.35
12	Sutton et al.		0.02 (0.00 - 0.22)	2.65
15	Wang et al.		0.40 (0.28 - 0.53)	20.68
16	Zhou et al.		0.46 (0.22 - 0.72)	11.41
	Overall (Male)*		0.45 (0.33 - 0.56)	
	**Overall (Total)***		0.50 (0.42 - 0.58)	

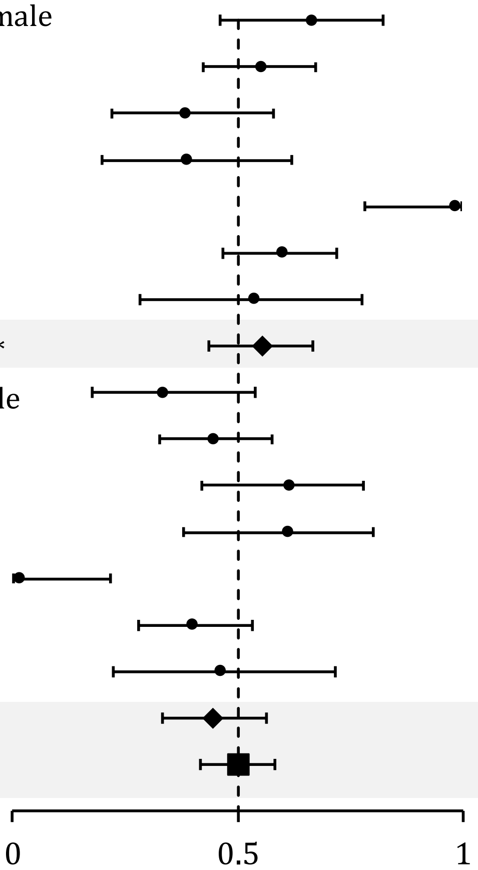

Heterogeneity: $I^2 = 58.9\%$, $\tau^2 = 0.222$, $p = 0.003$
*Random Model

Figure S1. Forest plot showing pooled prevalence of asymptomatic SARS-CoV-2 carriers according to gender

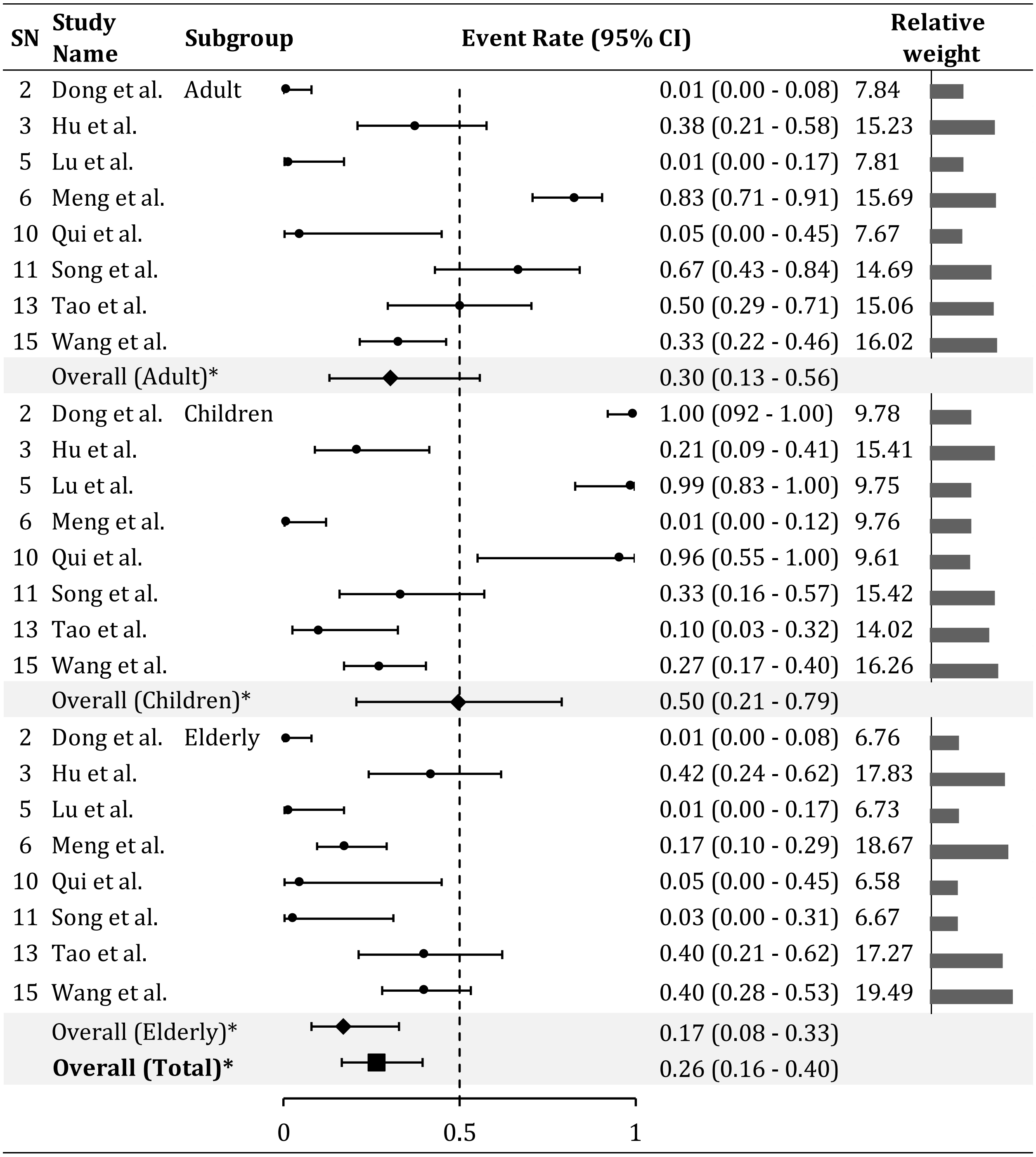

Heterogeneity: I^2 = 85.1%, τ^2 = 1.541, p 0.001

* Random Model

Figure S2. Forest plot showing pooled prevalence of asymptomatic SARS-CoV-2 carriers according to age

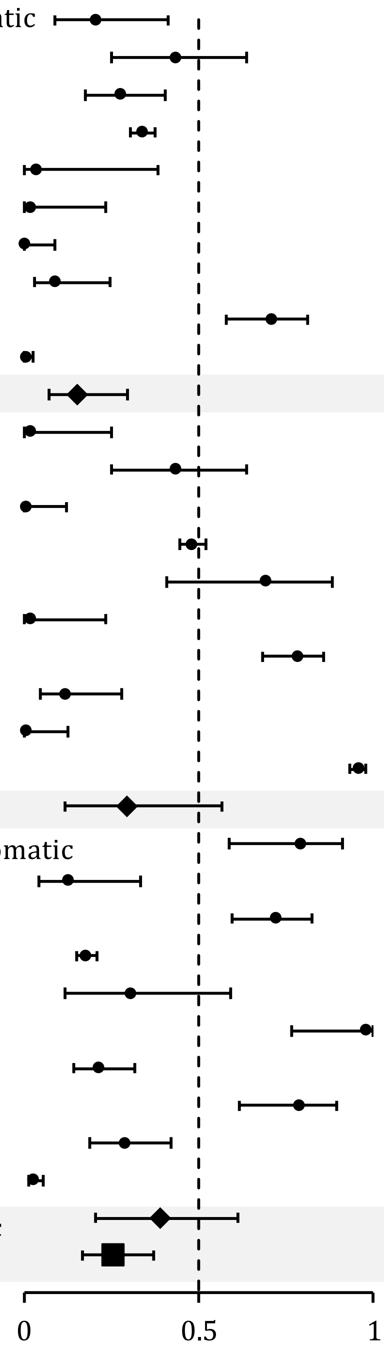

SN	Study Name	Outcome	Event Rates (95% CI)	Realtive weight
3	Hu et al.	Presymptomatic	0.21 (0.09 - 0.41)	11.4
4	Kimball et al.		0.44 (0.25 - 0.64)	11.95
6	Meng et al.		0.28 (0.18 - 0.40)	12.69
7	Mizumoto et al.		0.34 (0.30 - 0.38)	13.41
8	Nishiura et al.		0.04 (0.00 - 0.38)	5.4
9	Pan et al.		0.02 (0.00 - 0.24)	5.46
11	Song et al.		0.01 (0.00 - 0.09)	5.5
12	Sutton et al.		0.09 (0.03 - 0.25)	10.66
15	Wang et al.		0.71 (0.58 - 0.81)	12.67
16	Zhou et al.		0.01 (0.00 - 0.03)	10.85
	Overall Presymptomatic*		0.15 (0.07 - 0.30)	
3	Hu et al.	Symptomatic	0.02 (0.00 - 0.25)	7.15
4	Kimball et al.		0.44 (0.25 - 0.64)	11.81
6	Meng et al.		0.01 (0.00 - 0.12)	7.19
7	Mizumoto et al.		0.48 (0.44 - 0.52)	12.55
8	Nishiura et al.		0.69 (0.41 - 0.88)	11.09
9	Pan et al.		0.02 (0.00 - 0.24)	7.16
11	Song et al.		0.78 (0.68 - 0.86)	12.26
12	Sutton et al.		0.12 (0.05 - 0.28)	11.38
15	Wang et al.		0.01 (0.00 -0.13)	7.19
16	Zhou et al.		0.96 (0.93 - 0.98)	12.22
	Overall Symptomatic*		0.30 (0.12 - 0.57)	
3	Hu et al.	True asymptomatic	0.79 (0.59 - 0.91)	10.06
4	Kimball et al.		0.13 (0.04 - 0.34)	9.49
6	Meng et al.		0.72 (0.60 - 0.82)	10.91
7	Mizumoto et al.		0.18 (0.15 - 0.21)	11.34
8	Nishiura et al.		0.31 (0.12 - 0.59)	9.58
9	Pan et al.		0.98 (0.76 - 1.00)	5.5
11	Song et al.		0.22 (0.14 - 0.32)	10.99
12	Sutton et al.		0.79 (0.62 - 0.90)	10.41
15	Wang et al.		0.29 (0.19 - 0.42)	10.9
16	Zhou et al.		0.03 (0.02 - 0.06)	10.82
	Overall True asymptomatic		0.39 (0.20 - 0.61)	
	**Overall (Total)***		0.26 (0.17 - 0.37)	

Heterogeneity: I2 = 95.6%, τ2 = 1.467, p <0.001

* Random Model

Figure S3. Forest plot showing prevalence of COVID-19 clinical outcome among patients in included individual studies

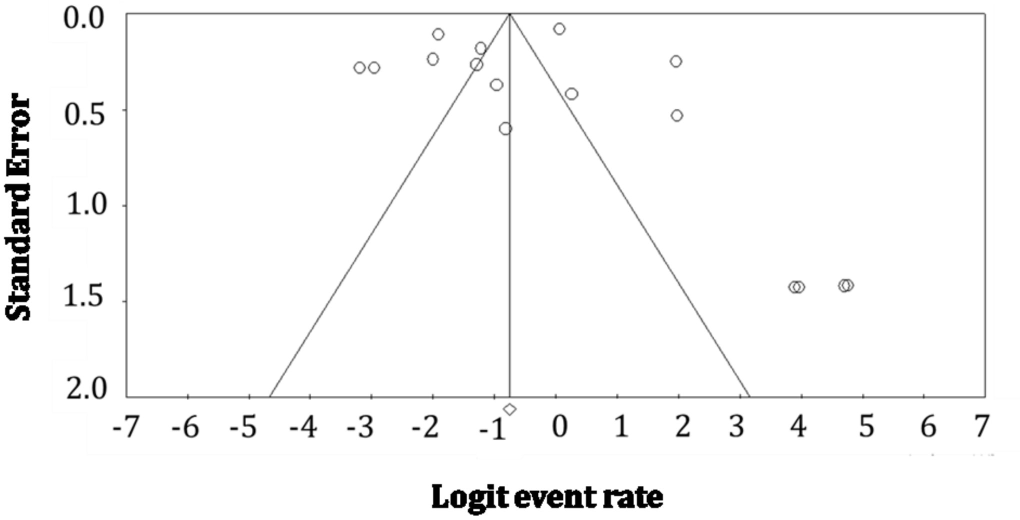

Figure S4. Funnel plot for assessing publication bias in Meta-analysis for prevalence of asymptomatic COVID-19 cases.
